# Characteristics of hypertension in the last 16 years in high prevalence region of China and the attribute ratios for cardiovascular mortality

**DOI:** 10.1186/s12889-022-14974-0

**Published:** 2023-01-16

**Authors:** Wenlong Zheng, Xiaohe Wang, Xiaodan Xue, Wei Li, Lili Fan, Shuang Zhang, Changkun Li, Zhuo Wang, Meiqiu Xie, Peng Xin, Guohong Jiang

**Affiliations:** 1 NCDs Preventive Department, Tianjin Centers for Disease Control and Prevention, No. 6 Huayue Road, Hedong District, Tianjin, 300011 China; 2 School of Public Health, Tianjin Medical University, Tianjin, China

**Keywords:** Hypertension, Epidemiological characteristics, Cardiovascular disease, Population attribution fractions

## Abstract

**Background:**

Tianjin is one of the cities with the highest prevalence of hypertension in China and one of the first regions to develop community management of hypertension. Our aim was to analyze the characteristics of hypertension in the last 16 years, and estimate the population attributable fraction for cardiovascular mortality in Tianjin, China.

**Methods:**

We compared the epidemiological characteristics of hypertension between 2002 and 2018 by analyzing data from the National Nutrition and Chronic Disease Risk Factor Survey. Subsequently, we obtained the cause-specific mortality in the same year from the Tianjin All Cause of Death Registration System (CDRS), and the population attributable fraction was used to estimate the annual cardiovascular disease (CVD) deaths caused by hypertension.

**Results:**

In 2002 and 2018, the crude prevalence, awareness, treatment rate in diagnosed, control rate in treated, and overall control rate of hypertension were 36.6% and 39.8%, 36.0% and 51.9%, 76.0% and 90.1%, 17.4% and 38.3%, 4.8% and 17.9%, respectively (*P* < 0.05). The mean SBP for males between the ages of 25 and 50 was significantly higher in 2018 than in 2002. The number of CVD deaths attributed to hypertension was 13.8 thousand in 2002 (account for 59.1% of total CVD deaths), and increased to 21.7 thousand in 2018 (account for 58.8% of total CVD deaths). The population attributable fraction have increased in the age groups of 25–44 and 75 and above, and decreased in the age group of 45–74 from 2002 to 2018.

**Conclusions:**

Compare to 2002, the proportion of CVD deaths attributed to hypertension remains high, particularly among younger and older people, despite a very significant increase in treatment and control rates for hypertension in 2018.

**Supplementary Information:**

The online version contains supplementary material available at 10.1186/s12889-022-14974-0.

## Background

High blood pressure (BP) is the leading cause of death globally and a core risk factor for cardiovascular disease (CVD). In 2017, 218 million (95% uncertainty interval [UI], 198–237 million) disability-adjusted life-years (DALYs) were attributed to high systolic blood pressure (SBP). [1] China has the largest population with hypertension and the highest total disease burden of hypertension in the world, characterized by high prevalence, low awareness, and low treatment and control rates. [1] Tianjin, a large city of over 10 million people in northern China, has one of the highest prevalence of hypertension in China, [2] and is also one of the first regions to carry out community management of hypertension. However, there were few reports on the prevalence characteristics, long-term trends of hypertension and its impact on cardiovascular disease mortality in Tianjin. Tianjin participated in the National Nutrition and Chronic Disease Risk Factors Survey [3] in 2002 and 2018 respectively. Using data from these two surveys, we analyzed the prevalence characteristics of hypertension and the trends over the last 16 years, and estimated the risk of cardiovascular mortality following the methodological framework and analytical strategy of the Global Burden of Disease (GBD) Study. [4].

## Methods

### Data source

Data of BP were obtained from the National Nutrition and Chronic Disease Risk Factors Survey in 2002 and 2018, including 2668 cases in 2002 and 4419 cases in 2018. The response rates were 94.3% in 2002 and 96.8% in 2018. The National Nutrition and Chronic Disease Risk Factors Survey is a nationally representative survey on the nutritional and health status of residents, organized by the National Health Commission using a multistage stratified cluster random sampling method at each point. [3, 5].

Data of cause of death in 2002 and 2018 were both obtained from the Tianjin All Cause of Death Registration System (CDRS), which was established and covered the entire population in 1984. [6] Garbage codes of cause of death were redistributed according to the well-established methods in the GBD study, [7] and all the data mapped age-, sex-, and cause-specific mortality. Based on the Comparative Risk Assessment in the GBD Study, Ischemic heart disease (IHD) (ICD-10 code: I20–I25), Ischemic stroke [ICD-10 code: I63, I65–I67 (except I67.4), I69.3], Hemorrhagic and other nonischemic stroke (ICD-10 code: I60–I62, I69.0–I69.2, I67.4), rheumatic heart disease (ICD-10 code: I01, I02.0, I05–I09), endocarditis, cardiomyopathy and myocarditis (ICD-10 code: I33, I40, I42), aorta aneurysm (ICD-10 code: I71), hypertensive heart disease (ICD-10 code: I11), atrial fibrillation, peripheral vascular disease, and other circulatory diseases [ICD-10 code: I48, I73, I70.2, I00, I02.9, I27–I28 (except I27.1), I30–I32 (except I31.2,I31.3), I34–I39, I47, I70.8, I72, I77–I80, I82–I84, I86–I98, G45] were identified as an etiological health outcomes of CVD associated with elevated BP. [7].

### BP measurements and definitions

In 2002, BP was measured with standard mercury sphygmomanometer (scale range 0–300 mmHg, 1 mmHg = 0.133 kPa) with an accuracy of 2 mmHg. Systolic and diastolic blood pressures were measured 3 times by the physician according to the Korotkoff sound, and the average of the last 2 was adopted in the final analysis. In 2018, BP was measured 3 times with a HEM-7071 OMRON (OMRON Corporation, Kyoto, Japan) electronic BP meter, and the average of the last 2 times was used for the final analysis.

The main outcomes were prevalence, awareness, treatment rate in diagnosed, control rate in treated, and overall control rate of hypertension. Hypertension was defined as SBP ≥ 140 mmHg or diastolic blood pressure (DBP) ≥ 90 mmHg and who self-reported on antihypertensive medication within 2 weeks and who report having been diagnosed with hypertension by a health professional, according to 2010 Chinese guidelines for the management of hypertension. [8] Awareness of hypertension was defined as the percentage of people with hypertension who self-reported being diagnosed with hypertension by a doctor. Treatment rate was defined as self-reported use of a prescription medication for hypertension within 2 weeks at the time of the interview in hypertension group. Control rate was defined as whose SBP < 140 mmHg and DBP < 90 mmHg in hypertension group.

Treatment rate in diagnosed was defined as the percentage of using antihypertensive medication in the past 2 weeks in patients with diagnosed hypertension.

1 $$treatment\;rate\;indiagnosed=\frac{\mathrm{number}\;\mathrm{of}\;\mathrm{treatment}}{\mathrm{number}\;of\;\mathrm{awareness}}\times100\%$$

Control rate in treated was the percentage of control (SBP < 140 mmHg and DBP < 90 mmHg) in treated group.

2 $$control\;rate\;intreated=\frac{\mathrm{number}\;\mathrm{of}\;\mathrm{control}}{\mathrm{number}\;\mathrm{of}\;\mathrm{treatment}}\times100\%$$

### Statistical analysis

Continuous variables were presented as means and 95% confidence intervals (CIs) and categorical variables were expressed as frequencies, percentages and proportions. The age-standardized rates were calculated by using the age composition of Segi’s world standard population. Pearson Chi-square was used to make between-group comparisons of categorical variables. SPSS 21.0 was used for statistical analyses. All *P*-values were derived from two-sided tests, and the significance level was set at *P* < 0.05.

We employed the comparative risk assessment framework to estimate the number of deaths for adults aged over 25 years attributable to hypertension in 2002 and 2018 in Tianjin, China. We first calculated the number of deaths by age and sex for each of the 11 types of cardiovascular diseases. Further, We obtained the relative risk (RR) for each 10 mmHg increase in SBP for each age group (25 years and older) for each health outcome from the GBD study, which used a meta-analysis based on randomized controlled trials and large cohort studies, and confounding factors were consistently adjusted. [7].

Population attributable fractions (PAFs) were calculated to obtain the proportion of CVD deaths attributable to elevated BP by comparing the observed SBP distribution with a theoretical minimum (mean of 115 mmHg with standard deviation (SD) of 6 mmHg) for each disease by sex and age group. The formula with BP treated as a continuous variable was:

3 $$PAF = \tfrac{{\int_{x = 0}^{m} {RR(x)P(x)dx - \int_{x = 0}^{m} {RR(x)P^{\prime}(x)dx} } }}{{\int_{x = 0}^{m} {RR(x)P(x)dx} }}$$

where RR(x) is the relative risk at SBP level x; P(x) is the observed population distribution of SBP; P’(x) is the theoretical minimum SBP distribution (mean of 115 mm Hg with SD of 6 mm Hg); and m is the maximum amount of SBP level. [7] CVD mortality attributable to elevated BP for each disease was calculated by multiplying the PAF with the observed deaths of CVD by sex and age group (≥ 25 years).

@RISK software (version 6.1 for Excel; Microsoft Corporation, Redmond, WA) was used to calculate uncertainty ranges. We defined risk-factor exposure levels and RR values as the input variables (RR as a lognormal distribution and others as normal distributions). The attributable fractions, attributable CVD deaths, were treated as the output variables. For each of the output variables, 95% uncertainty intervals were calculated using the 1000 iteration values generated between the 2.5th and 97.5th percentile.

## Results

### Characteristics of participants in 2002 and 2018

In 2002, there were 2668 participates, of whom 47.7% were male, 53.6% were from urban areas and 98.0% were Han ethnicity. The mean age was 46.54 ± 16.08. In 2018, there were 4419 participates, of whom 49.3% were male, 52.4% were from urban areas and 94.8% were Han ethnicity. The mean age was 48.67 ± 16.85. There were statistical significances of mean age, mean body mass index, percentage of Han ethnicity, and education attainment between 2002 and 2018 (Table 1).

**Table 1:** Characteristics of Study Population in 2002 and 2018.^a^

	**2002**	**2018**	***P-value***
No	2668	4419	
Age, year	46.5 ± 16.1	48.7 ± 16.8	< 0.001
Male, %	47.7%	49.3%	0.193
Urban area, %	53.6%	52.4%	0.332
Han ethnicity, %	98.0%	94.8%	< 0.001
Education attainment, %			
Primary school or lower	27.5%	22.1%	< 0.001
Junior high school	58.0%	54.5%	< 0.001
Senior high school or above	14.5%	23.4%	< 0.001
Body mass index, kg/m^2^	25.0 ± 5.0	25.9 ± 3.9	< 0.001

^a^Continuous variables were described with mean ± standard deviation; categorical variables were described with %

### Characteristics of the prevalence of hypertension in 2018

The crude prevalence, awareness rate, treatment rate, and overall control rate of hypertension were 39.8%, 51.9%, 46.8% and 17.9% among Tianjin residents in 2018, respectively. The treatment rate in diagnosed, control rate in treated were 90.1%, 38.3%. The prevalence of hypertension was higher in males (46.5%) than in females (33.2%), especially in the lower age-group, while the awareness was higher in females than in males. The prevalence, awareness rate was increasing by age. (Table 2).

**Table 2:** Prevalence, awareness, treatment, and control rates (%)of hypertension in Tianjin, China, 2018

Age-groups	N	Prevalence rate (95% CI)	Awareness rate (95% CI)	Treated rate in diagnose (95% CI)	Control rate in treated (95% CI)	Overall Control rate (95% CI)
**Whole participants**						
18–44	1950	19.8(18.0,21.5)	14.3(10.8,17.8)	78.2(67.3,89.1)	30.2(16.5,44.0)	3.4(1.6,5.2)
45–59	1210	46.0(43.2,48.8)	50.7(46.6,54.9)	90.1(86.6,93.6)	40.6(34.5,46.6)	18.5(15.3,21.8)
60-	1259	64.8(62.1,67.4)	70.6(67.8,73.4)	91.3(89.0,93.6)	37.8(33.7,42.0)	24.4(21.4,27.3)
Total	4419	39.8(38.3,41.2)	51.9(49.6,54.3)	90.1(88.2,92.1)	38.3(35.0,41.6)	17.9(16.1,19.7)
**Males**						
18–44	978	29.8(26.9,32.6)	11.0(7.4,14.6)	78.1(63.8,92.5)	36.0(17.2,54.8)	3.1(1.1,5.1)
45–59	600	53.0(49.0,57.0)	48.7(43.3,54.2)	91.0(86.5,95.5)	38.3(30.3,46.3)	17.0(12.9,21.1)
60-	602	67.3(63.5,71.0)	72.8(68.5,77.2)	89.5(86.0,93.0)	37.1(31.3,43.0)	24.2(20.0,28.4)
Total	2180	46.5(44.4,48.6)	47.5(44.5,50.6)	89.2(86.4,92.0)	37.4(32.9,42.0)	15.9(13.6,18.1)
**Females**						
18–44	972	9.7(7.8,11.5)	24.5(15.9,33.2)	78.3(61.4,95.1)	22.2(3.0,41.4)	4.3(0.2,8.3)
45–59	610	39.2(35.3,43.1)	53.1(46.8,59.5)	89.0(83.5,94.4)	43.4(34.2,52.5)	20.5(15.4,25.6)
60-	657	62.6(58.9,66.3)	68.4(63.9,72.9)	93.2(90.3,96.2)	38.6(32.7,44.4)	24.6(20.4,28.7)
Total	2239	33.2(31.3,35.2)	57.9(54.4,61.5)	91.2(88.5,93.9)	39.2(34.4,44.0)	20.7(17.8,23.6)

#### Comparison of characteristics of hypertension between 2018 and 2002

**Fig. 1:**
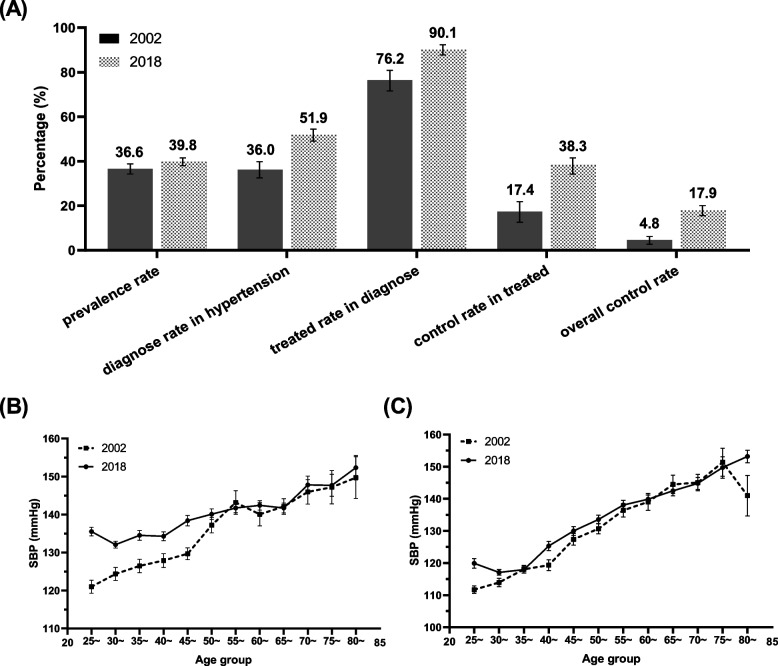
Comparison of hypertension characters between 2018 and 2002. **A** Prevalence, awareness, treatment, and control rates of hypertension; **B** SBP in males; **C** SBP in females. SBP, systolic blood pressure

The crude prevalence of hypertension was 39.8% in 2018, higher than in 2002 (36.6%) (^*2*^ = 6.5, *P* = 0.01). The crude awareness rate (51.9% vs 36.0%, ^*2*^ = 61.1, *P* = 0.000), treatment rate in diagnosed (90.1% vs 76.0%, ^*2*^ = 39.6, *P* = 0.000), control rate in treated (38.3% vs 17.4%, ^*2*^ = 37.2, *P* = 0.000)and overall control rate (17.9% vs 4.8%, ^*2*^ = 88.4, *P* = 0.000) all increased significantly in 2018 compared to 2002 (Fig. 1.A). For the age-adjusted rates, the prevalence (33.0% vs 32.0%, ^*2*^ = 14.4, *P* = 0.000), awareness rate (29.7% vs 24.5%, ^*2*^ = 469.4, *P* = 0.000), treatment rate in diagnosed (69.7% vs 58.5%, ^*2*^ = 1871.4, *P* = 0.000), control rate in treated (25.2% vs 21.3%, ^*2*^ = 285.9, *P* = 0.000) and overall control rate (9.6% vs 3.4%, ^*2*^ = 2226.5, *P* = 0.000) also increased significantly in 2018 compared to 2002.

In 2018, the mean SBP was significantly higher in 2018 than in 2002 for males between the ages of 25 and 50, and that was basically the same for males over 50 years (Fig. 1.B). The mean SBP was higher in 2018 than in 2002 for females in the age groups of 25–29, 30–34, 40–44, and 80 and older (Fig. 1.C).

#### CVD deaths attributed to hypertension in 2002 and 2018

In 2002, 13.8 thousand CVD deaths (account for 59.1% of total CVD deaths) were attributed to hypertension, including 5.7 thousand IHD, 7.7 thousand stroke and 0.4 thousand other CVDs. In 2018, a total of 21.7 thousand CVD deaths were attributed to hypertension, including 11.8 thousand IHD, 9.7 thousand strokes and 0.2 thousand other CVDs, which accounts for 58.8% of total CVD deaths. Both attributing number and ratio of male’s were higher than female’s (12.0 thousand versus 9.8 thousand, 60.1% versus 57.5%) (Table 3).

**Table 3:** CVDs deaths attribute to hypertension in 2018 and 2002 in Tianjin, China

health outcomes	Male		Female		Both sexes	
	Deaths No.(95%UI) Thousands	Death PAFs,%(95% UL)	Deaths No.(95%UI) Thousands	Death PAFs,%(95% UL)	Deaths No.(95%UI) Thousands	Death PAFs,%(95% UL)
2018						
Ischemic heart disease	6.1(5893–6188)	61.7%(58.6–62.7)	5.7(5689–5842)	59.5%(58.3–59.9)	11.8 (11353–12205)	60.5%(59.3–63.5)
Ischemic stroke	3.9(3857–4003)	56.7%(56.5–59.6)	2.8(2793–2927)	53.5%(51.3–53.8)	6.7(6546–6701)	55.2%(54.9–56.3)
Hemorrhagic stroke	1.8(1797–1838)	66.3%(64.4–66.7)	1.2(1169–1216)	62.6%(61.5–63.2)	3.0(2991–3076)	64.43%(64.0–65.1)
other CVDs	0.1(133–139)	35.2%(34.4–35.3)	0.1(92–98)	31.4%(30.7–31.9)	0.2(229–235)	33.5%(33.4–34.0)
all CVDs	12.0(11941–12438)	60.1%(59.1–60.1)	9.8(9682–9889)	57.5%(55.9–57.7)	21.7(21201–21837)	58.8%(58.0–59.7)
2002						
Ischemic heart disease	3.1(3111–3293)	61.4%(60.7–64.3)	2.6(2573–2751)	58.4%(57.6–60.1)	5.7(5696–6091)	60.2%(59.9–61.3)
Ischemic stroke	2.2(2181–2322)	58.9%(58.2–59.8)	1.6(1591–1677)	56.1%(55.3–57.3)	3.8(3808–3936)	57.6%(55.1–58.0)
Hemorrhagic stroke	2.3(2299–2382)	65.9%(65.8–68.1)	1.6(1574–1608)	63.1%(62.8–64.2)	3.9(3882–4020)	64.8%(63.9–65.3)
other CVDs	0.2(188–192)	33.4%(32.5–34.2)	0.2(199–209)	29.6%(29.2–29.9)	0.4(380–394)	31.1%(30.4–31.7)
all CVDs	7.8(7606–7913)	60.4%(60.0–61.1)	6.0(5908–6036)	57.2%(56.8–57.6)	13.8(13611–13948)	59.1%(58.9–59.4)

**Fig. 2:**
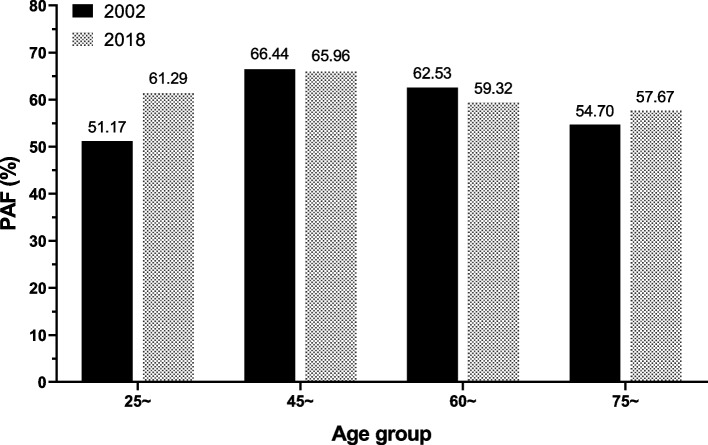
Cardiovascular disease deaths attributed to hypertension in 2002 and 2018 according to age groups.PAF, population-attributable fraction

In 2002 and 2018, the attribution ratios were 51.2% and 61.3%, 66.5% and 66.0%, 62.5% and 59.3%, and 54.7% and 57.7% for the age groups of 25–44, 45–59, 60–74, and 75 years and older, respectively. The attribution ratios have increased in the age groups of 25–44 and 75 and above, and decreased in the age groups of 45–74 from 2002 to 2018 (Fig. 2). The attributing number of 75 and above age group was extremely higher in 2018 than in 2002 (13.7 thousand vs 5.9 thousand).

## Discussion

In this study, we found a high prevalence of hypertension and a large attribution to the burden of CVD, although the awareness, treatment, and control rates of hypertension have improved tremendously over the past 16 years in Tianjin, China.

Our results showed that the prevalence of hypertension in Tianjin in 2018 was much higher than the national level (23.2%, from 2012 to 2015). [2] Previous studies have shown that the higher incidence and prevalence of hypertension in northeastern and eastern China compared to western China [2, 9] may be closely associated with higher salt intake, obesity rates and higher body weight. In 2017, high sodium intake was the leading dietary risk for deaths and high DALYs in China. [10] About 98.7% of residents consume more than 6 g/day of salt, which was 3–4 times higher than the average standard in Tianjin in 2015. [11] Besides, Tianjin also has the highest obesity rate in China, with an adult obesity rate of 12.2%, [12] which is also a major risk factor for hypertension.

The study showed that the awareness, treatment and control rates of hypertension among Tianjin residents have improved significantly, although the prevalence of hypertension among Tianjin residents has shown a slight upward trend over the past 16 years, which was consistent with previous studies [2, 13–15]. Over the past 20 years, the prevalence of hypertension had declined in some high-income countries, [16–19] but had increased in low- and middle-income countries, including China, [13–15] which largely due to the prevalence of unhealthy lifestyles as a result of rapid economic development and the acceleration of the aging process. [2] Comparing the population characteristics of the two surveys, we found that the mean age, mean BMI and education level were all higher in 2018 than in 2002, which may partly explain the changes in the prevalence characteristics of hypertension. The prevalence of hypertension was higher in the elderly and in people with high BMI, while higher levels of education were usually accompanied by higher levels of awareness.

However, the increase of awareness, treatment, and control rates may be closely related to the improvement of community public health service level. Since 2007, China has gradually incorporated the management of hypertensive patients into community public health services. [20, 21] Community doctors provide free follow-up management for patients four times a year. At present, over 90% of patients with hypertension and diabetes use community public health services. [22] Another reason may be related to the significant improvement of medical insurance coverage rate. [23] The medical insurance coverage rate in China was only 10% in 2003, but it raised to 96.7% by 2019. [24] This could provide more medical insurance for residents to manage their hypertension. However, the awareness and control rates of hypertension among Tianjin residents were still far below that of developed countries, [25] especially among the 18–45 age group, where the awareness rate was only 14.25%, compared to 74.7% in the United States. [26].

Although the treatment rate in diagnosed to be a high level, the control rate is still relatively low. Fixed-dose combination therapy can help to improve the control rate of treated hypertensive patients. [27] However, the proportion of single-drug use in Tianjin was 62.97%, and the proportion of combination drugs was lower, even in patients with grade 2 and 3 hypertension. [28] So, we should promote the “National Clinical Practice Guidelines on the Management of Hypertension”, [29] especially in community public health services.

A new finding in our study was that the mean SBP in males increased most in the 25–50 age-group, but the awareness rate was lower, a phenomenon that needs to be given adequate attention. This may be mainly due to the prevalence of higher BMI, lower physical activity levels, higher red meat intake, [30] smoking, excessive alcohol consumption, and higher dietary sodium intake in males. [10, 31, 32]. A study showed that elevated SBP was a major predictor of CVD events regardless of whether diagnostic criteria for hypertension were met. [33] Previous studies have shown that the incidence of acute myocardial infarction [34] and the mortality of cerebral infarction [35] in males in the lower age group in Tianjin tended to increase, while in females it was relatively stable, which is basically consistent with the trend of BP changes in this study. The risk of death from CVD was also higher in the lower age group than in the higher age group in patients with hypertension. [36, 37] People in the lower age group usually pay little attention to BP, and the current public health policy in China mainly emphasizes in clinical practice that people aged 35 and above should have their BP checked first, which may be one of the important reasons for the low awareness rate. Therefore, more active hypertension prevention and control strategies should be implemented in the lower age group. Health education and BP examination should be carried out for the lower age group and the frequency of BP measurement should be increased through community service or family self-testing.

Another new finding of our study was an increase in the attribution ratio of CVD attributable to high SBP in the 25–44 and 75 and above age groups in Tianjin. CVD is the largest single contributor to global mortality, [38] which accounts for more than 40% of deaths in China [39] and 53.7% in Tianjin city. [40] Our results showed a significant increase in the number and proportion of deaths attributed to hypertension in the age group 75 years and older compared to other age groups from 2002 to 2018. Although there is still controversy over the target of BP control in the elderly, [41, 42] cohort studies from China have shown that all-cause mortality and CVD mortality were significantly increased in elderly when their SBP is ≥ 160 mmHg. [43–45] Control of hypertension is the most important measure to reduce the DALYs of CVD. [46].

In 2018, the number of deaths from ischemic heart disease exceeded that of stroke attributable to hypertension. This was associated with an increase in age-standardized mortality from ischemic heart disease and a decrease in age-standardized mortality from stroke. [4, 47] The factors contributing to these declines mainly include improvements in healthcare coverage, upgrades in medical technology and improvements in the public health environment for stroke prevention by the government. [48].

Some limitations cannot be ignored. One of the study’s limitations is that the number of survey samples in 2002 was significantly smaller than that in 2018. Although it could meet the requirement of minimum representative sample size in 2002, the results of stratified analysis may be unstable. Furthermore, the RRs of SBP to CVD were from the GBD study, which was a global meta-result and not the real situation of Tianjin. More cohort studies are needed in the future.

## Conclusions

Although the treatment and control rates of hypertension increased a lot, the prevalence of hypertension and the proportion of CVD deaths attributed to hypertension remains high especially in younger and old age groups, suggesting that effective hypertension prevention measures should be targeted to reduce the overall prevalence of hypertension according to different regions and populations. The results of this study provide a scientific basis for the development of relevant hypertension prevention and control policies. In addition, large population-based prospective studies should be implemented to explore effective preventive measures to reduce the incidence and prevalence of hypertension and thereby reduce the risk of cardiovascular mortality in the population.

## Supplementary Information

**Additional file 1.**

## Data Availability

The data that support the findings of this study are available from the corresponding author but restrictions apply to the availability of these data, which were used under license for the current study, and so are not publicly available.Data are however available from the authors upon reasonable request and with permission of the Tianjin Center for Disease Control and Prevention ethics committees.

## Abbreviations

BP: Blood pressure
CVD: Cardiovascular disease
UI: Uncertainty interval
DALYs: Disability-adjusted life-years
SBP: Systolic blood pressure
GBD: Global Burden of Disease
CDRS: Tianjin All Cause of Death Registration System
ICD-10: International Classification of Diseases 10th Revision
IHD: Ischemic heart disease
DBP: Diastolic blood pressure
PAFs: Population-attributable fractions
SD: Standard deviation
