# Supplementary material for: Characteristics of hypertension in the last 16 years in high prevalence region of China and the attribute ratios for cardiovascular mortality
Source: BMC Public Health. 2023 Jan 16;23:114. doi: 10.1186/s12889-022-14974-0 (PMC9841650; doi:10.1186/s12889-022-14974-0)
Supplement: Supplementary file 1 — Additional file 1. [file 12889_2022_14974_MOESM1_ESM.docx]

Supplementary table 1 the RR (95 %CI) per 10mmHg increase in SBP by age(y) group for related cardiovascular diseases

| Health outcomes | 25-29 | 30-34 | 35-39 | 40-44 | 45-49 | 50-54 | 55-59 | 60-64 | 65-69 | 70-74 | 75-79 | 80+ |
| --- | --- | --- | --- | --- | --- | --- | --- | --- | --- | --- | --- | --- |
| rheumatic heart disease | 1.63(1.17-2.31) | 1.47(1.17-1.90) | 1.32(1.14-1.58) | 1.23(1.09-1.42) | 1.21(1.10-1.37) | 1.19(1.11-1.32) | 1.18(1.10-1.29) | 1.16(1.09-1.27) | 1.14(1.06-1.25) | 1.13(1.05-1.24) | 1.12(1.06-1.24) | 1.10(1.04-1.28) |
| ischemic heart disease | 1.97(1.44-2.60) | 1.82(1.46-2.21) | 1.67(1.46-1.91) | 1.57(1.40-1.80) | 1.53(1.39-1.71) | 1.49(1.39-1.62) | 1.45(1.37-1.54) | 1.41(1.33-1.49) | 1.36(1.26-1.46) | 1.33(1.22-1.42) | 1.30(1.23-1.40) | 1.27(1.13-1.44) |
| ischemic stroke | 1.85(1.39-2.59) | 1.77(1.43-2.25) | 1.69(1.40-2.04) | 1.63(1.35-1.95) | 1.57(1.36-1.83) | 1.52(1.36-1.70) | 1.47(1.34-1.60) | 1.41(1.30-1.52) | 1.36(1.21-1.49) | 1.32(1.17-1.45) | 1.28(1.18-1.39) | 1.20(1.11-1.37) |
| hemorrhagic stroke | 2.13(1.55-2.92) | 2.05(1.59-2.66) | 1.97(1.59-2.47) | 1.87(1.49-2.30) | 1.78(1.48-2.12) | 1.68(1.45-1.93) | 1.58(1.40-1.76) | 1.48(1.33-1.62) | 1.38(1.21-1.54) | 1.32(1.16-1.50) | 1.31(1.19-1.45) | 1.28(1.13-1.52) |
| endocarditis, myocarditis and myocardial disease | 1.76(1.27-2.42) | 1.61(1.29-2.01) | 1.46(1.28-1.64) | 1.37(1.23-1.51) | 1.34(1.22-1.45) | 1.31(1.22-1.39) | 1.28(1.21-1.34) | 1.25(1.18-1.30) | 1.22(1.13-1.29) | 1.19(1.12-1.26) | 1.18(1.12-1.24) | 1.13(1.07-1.24) |
| atrial fibrillation and peripheral neuropathy and other circulatory diseases | 1.76(1.34-2.43) | 1.63(1.38-2.03) | 1.50(1.40-1.64) | 1.42(1.34-1.51) | 1.39(1.33-1.46) | 1.36(1.31-1.41) | 1.33(1.29-1.37) | 1.30(1.27-1.33) | 1.27(1.23-1.31) | 1.24(1.20-1.28) | 1.21(1.18-1.24) | 1.13(1.09-1.19) |
| aortic aneurysm | 1.54(1.26-2.17) | 1.47(1.29-1.82) | 1.39(1.30-1.54) | 1.35(1.23-1.45) | 1.32(1.23-1.41) | 1.30(1.23-1.36) | 1.27(1.22-1.33) | 1.25(1.19-1.30) | 1.22(1.16-1.29) | 1.20(1.14-1.26) | 1.18(1.13-1.23) | 1.12(1.07-1.19) |
